# Impact of Plasma Exposure of Statins and Their Metabolites With Major Adverse Cardiovascular Events in Chinese Patients With Coronary Artery Disease

**DOI:** 10.3389/fphar.2020.00675

**Published:** 2020-05-27

**Authors:** Xiao-hong Zhou, Li-yun Cai, Wei-Hua Lai, Xue Bai, Yi-bin Liu, Qian Zhu, Guo-dong He, Ji-Yan Chen, Min Huang, Zhi-ling Zhou, Shi-long Zhong

**Affiliations:** ^1^Guangdong Provincial Key Laboratory of Coronary Heart Disease Prevention, Guangdong Cardiovascular Institute, Guangdong Provincial People's Hospital, Guangdong Academy of Medical Sciences, Guangzhou, China; ^2^Department of Pharmacy, Guangdong Provincial People's Hospital, Guangdong Academy of Medical Sciences, Guangzhou, China; ^3^School of Pharmaceutical Sciences, Southern Medical University, Guangzhou, China; ^4^Laboratory of Drug Metabolism and Pharmacokinetics, School of Pharmaceutical Sciences, Sun Yat-sen University, Guangzhou, China; ^5^Department of Pharmacy, Zhuhai People’s Hospital (Zhuhai Hospital affiliated with Jinan University), Zhuhai, China

**Keywords:** major adverse cardiovascular events, death, atorvastatin, rosuvastatin, metabolites, plasma exposure

## Abstract

The selection of optimum statin intensity is inconclusive, and the association of plasma exposure of statins and metabolites with major adverse cardiovascular events (MACEs) is unclear. This study sought to compare the effect of low (quartile 1), intermediate (quartiles 2 and 3), and high (quartile 4) plasma exposure of statins and metabolites on MACE, re-ischemia events and death in patients with coronary artery disease (CAD) at 5 years. A total of 1,644 patients in atorvastatin (AT) cohort and 804 patients in rosuvastatin (RST) cohort were included, and their plasma concentration of statins and metabolites was categorized as low-, mid-, or high-group. The association between the plasma levels of statins and metabolites and the incidence of primary endpoint in patients was assessed by Cox proportional hazard models. Intensive AT exposure (Q4 > 5.32 ng/ml) was significantly associated with increased risk of death compared with low (hazard ratio [HR]: 1.522; 95% confidence interval [CI]: 1.035–1.061; P = 0.0022) or moderate exposure (HR: 2.054; 95% CI: 1.348–3.130; P = 0.0008). This association was also found in AT’s five metabolites (all P < 0.01). In patients with RST treatment, moderate RST concentration (0.53–4.29 ng/ml) *versus* low concentration had a significantly lower risk of MACE and re-ischemia events. (HR: 0.532, 95% CI: 0.347–0.815, P = 0.0061 and HR: 0.505, 95% CI: 0.310–0.823, P = 0.0061, respectively). A higher plasma exposure of AT and metabolites has a significantly higher risk of death, and moderate RST exposure has a significantly lower risk of MACE and re-ischemia events in Chinese patients with CAD. The harms of high plasma exposure should be considered when prescribing statins to patients because it may be a risk factor for having poor prognosis in patients with CAD.

## Clinical Perspective

### What Is New?

- To our knowledge, our study is the first to evaluate the effect of the plasma exposure of statin and metabolites on cardiovascular events in Chinese patients with CAD. We found that a higher plasma exposure of AT and metabolites has a significantly higher risk of death.
- The moderate RST exposure has a significantly lower risk of MACE and re-ischemia events.

### Implications

- In this study, patients taking moderate-intensity statins (10 mg of rosuvastatin, 20 mg of atorvastatin) represented 87% of all patients, and they are subject to a high inter-individual variability in plasma concentration. The pharmacokinetics of statins might therefore be a critical parameter for its activity.
- The following two metabolites may be good predictive biomarkers of cardiovascular outcomes: the active metabolite of atorvastatin, 4-AT, which is independently associated with increased risk of death, and the inactive metabolite 4-ATL, which is independently associated with high risk of MACE.
- Compared with low-plasma statin exposure, moderate exposure could achieve equal or better efficacy in terms of composite cardiovascular events, whereas high exposure of statin metabolites might increase the risk of death.

## Introduction

Statin therapy is extensively used in the primary and secondary prevention of coronary artery disease (CAD). It is effective in reducing the rates of major adverse cardiovascular events (MACEs) by approximately a quarter and improving long-term survival compared with placeb (Cholesterol Treatment Trialists C, et al., 2010; Fitchett et al., 2015; Silverman et al., 2016).

Intensive or high-dose statin therapy has been proposed to increase therapeutic benefit. Although numerous reports indicated that intensive statin therapy could improve the clinical outcomes (LaRosa et al., 2005; Pedersen et al., 2005; Gibson et al., 2009), no direct evidence is available to guide the selection of optimum intensity for initiating statins, and high-dose statin therapy may not be as beneficial as expected. Currently, a large observational cohort study suggested that the mortality and hospital admission rates for adverse cardiovascular events at 1 year were similar between residents taking intensive-dose statins and those taking moderate-dose statins (Campitelli et al., 2019). A systematic review for the US Preventive Services Task Force (USPSTF) showed that risk estimates for statins *versus* placebo for all-cause mortality were similar in trials of low-, moderate-, and high-intensity statins (Chou et al., 2016). An additional randomized trial even revealed that high-dose statin pretreatment before percutaneous coronary intervention did not reduce MACEs compared with low-dose statin pretreatment (Kim et al., 2010). Moreover, no significant difference was found in MACE between patients with high-dose therapy and those with mid-dose therapy after coronary artery bypass graft surgery (Kulik et al., 2019). In addition, high-dose statin therapy may be associated with increased risks of acute kidney injury, myopathy, gastrointestinal hemorrhage, and diabetes (Group et al., 2008; Ridker et al., 2008; Dormuth et al., 2013; Martinez et al., 2019), thereby increasing the risk of cardiovascular events.

Considering that important evidence gaps persist, recommendations were varied among guidelines (Stone et al., 2014; Catapano et al., 2016; Force et al., 2016). For example, the USPSTF guideline (Force et al., 2016) recommends to initiate low- to moderate-dose statin treatment for adults aged 40–75 years who have no history of cardiovascular disease (CVD), have one or more CVD risk factors, and have a calculated 10-year CVD event risk of 10% or greater. Meanwhile, the American College of Cardiology/American Heart Association guideline (Stone et al., 2014) recommends moderate- to high-dose statins for most asymptomatic adults aged 40–75 years without CVD history and who have a low-density lipoprotein cholesterol (LDL-C) concentration of 190 mg/dl or greater, diabetes, or an estimated 10-year CVD event risk of 7.5% or greater.

In the absence of clear consensus on high-intensity statin treatment, adopting high-dose statins for Asian patients should be of a greater concern, given that Asians can tolerate a higher-plasma statin concentration for a given dose compared with Caucasians (Lee et al., 2005; Liao, 2007). Considering that the therapeutic response at a given dose is highly variable between individuals (Pedro-Botet et al., 2001), using plasma concentration to predict therapeutic effect and further applying stratified concentrations (low, moderate, and high concentrations) to evaluate the risk of MACEs among patients should be more accurate than dosage.

Therefore, in this study, we quantitatively analyzed the plasma exposure of two widely prescribed statins, namely, atorvastatin (AT) and rosuvastatin (RST), and their metabolites. Then, we assessed the impact of high-statin concentrations on the occurrence of MACE, re-ischemia events, and death in 2,448 Chinese patients with CAD.

## Methods

### Ethics Statement

The present study was approved by the Medical Ethical Review Committee of Guangdong General Hospital and conducted according to the Declaration of Helsinki. Written Informed consent was obtained from all individual participants included in the study.

### Study Design and Patients

We conducted a prospective two-stage study to evaluate the effect of two statins on MACE, re-ischemia events, and death separately.

All patients were sequentially prospectively enrolled in Guangdong General Hospital between January 2010 and December 2013 according to the same inclusion and exclusion criteria. Baseline information, including demographics, medical history, biochemical measurements, and medication was obtained from the hospital information database. Cardiac Surgery (SYNTAX) score based on the results of coronary angiography was calculated by two experienced interventional cardiologists on the website http://www.syntaxscore.com.

### Patient Recruitment

The inclusion criterion was patients with coronary heart disease confirmed by coronary angiography (≥50% vascular stenosis). The exclusion criteria included the following: (1) pretreatment with other statins in 2 weeks; (2) age <18 years or >80 years; (3) renal insufficiency (serum creatinine [CREA] concentration > 3 times the upper limit of the normal value [345 μmol/L], renal transplantation, or dialysis); (4) liver insufficiency (serum transaminase concentration > 3 times the upper limit of the normal value [120 U/L], or a diagnosis of cirrhosis); (5) being pregnant or lactating; (6) advanced cancer or hemodialysis; (7) the concentrations of statins or their metabolites were lower than the limit of detection (3:1 noise); and (8) incomplete information about cardiovascular events during follow-up.

### SYNTAX Scoring

For the measurement of Cardiac Surgery (SYNTAX), images of coronary angiography were obtained using the Syngo Dynamics cardiovascular imaging software (Siemens Medical Solutions USA, Inc., Malvern, Pennsylvania), and SYNTAX score was calculated per method, as described on the website http://www.syntaxscore. The SYNTAX score accurately represents the severity of CVD and describes the characteristics of coronary lesions, including coronary dominance, number of diseased vessels, degree of vascular disease, and lesion characteristics.

The clinician selects Yes or No in turn, on the SYNTAX scoring software (SYNTAX Score Calculator), with reference to coronary angiography data. The software automatically scores each lesion and then accumulates the total score as the SYNTAX score. The SYNTAX score is divided into SYNTAX low < 22 points, SYNTAX median = 22–32 points, and SYNTAX high > 32 points.

### Study Endpoints

The study endpoints included MACE, re-ischemia events, and death, representing the therapeutic efficacy of statins. MACE was defined as the occurrence of cardiac death, nonfatal myocardial infarctions, coronary revascularization, and cerebral infarction. Re-ischemia events were defined as nonfatal repeated myocardial infarctions, repeated revascularization, and cerebral infarction.

All participants were followed prospectively for the study’s endpoints. Follow-up information was collected based on inpatient and outpatient hospital visits and telephone contacts with the patients or their families until April 2017. At each follow-up assessment (every 6 months), the participants were questioned about new, adverse cardiovascular events. Baseline risk factors and medication use were recorded for the enrolled patients.

### Biochemical Parameter Measurement and Plasma Sample Preparation

Alanine aminotransferase (ALT), aspartate aminotransferase (AST), CREA, cholesterol (CHOL), creatine kinase (CK), creatine kinase MB (CKMB), apolipoprotein a (APOA), and other standard clinical parameters were determined by biochemical methods on the second day of each patient’s admission.

Each eligible patient had been consuming the same dose of AT or RST for at least 7 days prior to blood sampling. Statin dosage was prescribed by a physician in accordance with patients’ condition. Blood samples were obtained at 10–12 h postdose and collected in EDTA-coated tubes. Plasma was separated within 2 h by centrifugation at 3,000 rpm for 10 min at 4°C and then stored at −80°C until analysis.

### Quantification of Statins and Their Metabolite Concentrations in Plasma

AT and its five metabolites, namely, 2-hydroxy atorvastatin (2-AT), 4-hydroxy atorvastatin (4-AT), atorvastatin lactone (ATL), 2-hydroxy atorvastatin lactone (2-ATL), and 4-hydroxy atorvastatin lactone (4-ATL), in human plasma were quantified according to our published method (Cai et al., 2017).

A sensitive UPLC-MS/MS assay for the simultaneous quantification of RST, *N*-desmethyl rosuvastatin (DM-RST), and rosuvastatin lactone (RSTL) in human plasma was also developed and validated. The analytes and internal standard (carbamazepine) were extracted from 200 μl of buffered human plasma (adding 100 μl of ammonium acetate of pH 4.0 to 100 μl of human plasma) by liquid–liquid extraction with ethyl acetate and then separated on the ACQUITY UPLC HSS T3 column (3.0 mm × 100 mm, 1.8 μm). Then, they were eluted at 0.3 ml/min by using a mobile phase containing acetonitrile and 0.05% of formic acid in water over a linear gradient of 30–85% of acetonitrile. Mass was detected on the Waters Xevo TQ-S triple-quadrupole mass spectrometer in a positive electrospray ionization mode. The responses of RST, RSTL, and DM-RST were optimized at the m/z 482.1→258.1, 464.1→270.1, 468.0→258.0, respectively. RST, RSTL, DM-RST in human plasma were quantified according to our published method (Bai et al., 2018).

### Statistical Analysis

Demographic and clinical characteristics were summarized using counts (percentages) for categorical variables and mean (standard deviation, SD) for continuous variables. The concentrations of statins and metabolites were log-transformed to meet the assumption of normality prior to analysis. Spearman correlation coefficients were calculated to describe the correlations between plasma concentration of statins and their metabolites.

To identify the contributing factors of statin and metabolite concentration, we performed linear regression analysis. We then applied univariable and multivariable Cox regression analyses to evaluate the effects of plasma concentrations, baseline demographic, and clinical characteristics on the risk of MACE, re-ischemia event, and death and to estimate hazard ratios (HRs) and 95% confidence intervals (CIs). Variables with P < 0.05 were entered into the multivariable model, and only variables with P < 0.05 were retained in the model. P < 0.05 was considered statistically significant. The method of selection is forward stepwise.

To further investigate whether low or high statin concentration can affect the risks of MACE, re-ischemia event, and death, we stratified patients into three groups, which were those with concentrations lower than the first quartile (<Q1), concentrations in the interquartile range (≥Ql and ≤Q3), and concentrations higher than the third quartile (> Q3). These three groups represented low, moderate, and high concentrations, respectively. The proportion of patients in each group at different dosages was calculated to describe the plasma-concentration distribution of statins and their metabolites for the certain dose. Cox regression analysis was also conducted to compare the difference in the occurrence of MACE, re-ischemia events, and death in patients stratified by the plasma concentration of statins and their metabolites. Variables with P < 0.0167 (P = 0.05/3 comparisons) were considered statistically significant. Cumulative incidence of the endpoints was visualized using the Kaplan–Meier method. Data were analyzed using R (version 3.4.3, http://www.R-project.org/) and SAS 9.4 (SAS Institute, Cary, NC, USA).

## Results

### Patient Characteristics and the Distribution of Plasma Exposure of AT and RST in Patients

**Figure 1:**
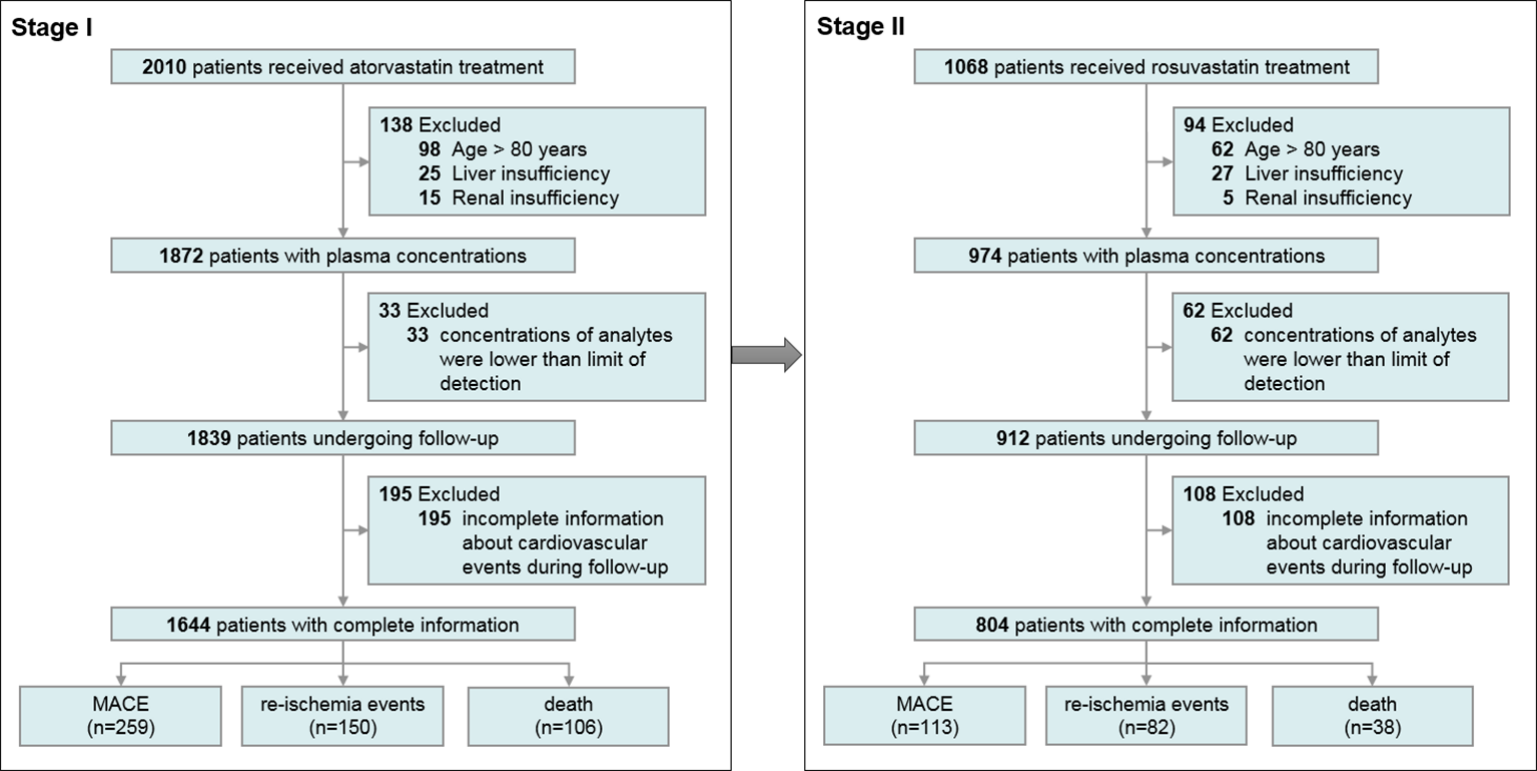
Flow chart of the enrolment of the participants.

We recruited 1,644 patients taking AT in stage I to evaluate the impact of statin exposure on MACE, re-ischemia, and death, and 804 patients taking RST in stage II to further confirm the idea. An overview of the enrollment of the patients is presented in **Figure 1**.

In stage I, 87.31% received the drug at the same dose of 20 mg, but a high interpatient variability for AT was found. The distribution of plasma concentrations at different doses is summarized in **Supplementary Table S1**.The plasma exposure of AT was widely varied, with a plasma AT concentration of 0.2–40 ng/ml (Q1 = 1.20 ng/ml, Q3 = 5.32 ng/ml) at 10–12 h postdose. The concentrations of five metabolites highly correlated with AT concentration (all r > 0.5, P < 0.0001, **Supplementary Figure S1**). Patients’ baseline characteristics and their impact on the AT concentration are summarized in **Supplementary Tables S3 and S4**. As expected, older age (estimate = 0.0140, P = 0.0002), higher dosage (estimate = 0.0219, P < 0.0001), higher ALT level (estimate = 0.0059, P = 0.0041), higher CREA (estimate = 0.0026, P = 0.0195), high LDLC level (estimate = 0.1199, P = 0.0016), and low APOA (estimate = −0.4192, P = 0.0011) were independently associated with a higher AT concentration (**Supplementary Table S3**). Hence, the abnormality of hepatic and renal function could significantly increase systemic AT exposure.

In stage II, most patients consumed moderate-intensity RST (10 mg), and the proportion was 87.31%. RST and metabolite exposure have high inter-individual variations (**Supplementary Table S2**). The plasma exposure of RST was widely varied, with a plasma RST concentration of 0.1–50 ng/ml (Q1 = 0.53 ng/ml, Q3 = 4.29 ng/ml) at 10–12 h postdose. The metabolites of DM-RST and RSTL concentrations in plasma were highly associated with RST concentration (r = 0.7790 and 0.7412, respectively; **Supplementary Figure S2**). Patients’ baseline characteristics and their impact on the RST concentration are summarized in **Supplementary Table S5**. Univariate and multivariate linear regression analyses revealed that plasma RST concentration was lower in patients with a higher CK level (estimate = −0.0011, P = 0.0005), higher LDLC level (estimate = −0.2589, P = 0.0005) and taking calcium channel blockers (estimate = −0.4789, P = 0.0055) (**Supplementary Table S5**).

### Impact of AT and Its Metabolite Exposure on MACE, Re-Ischemia Events, and Death

We observed that 259, 150, and 106 patients experienced MACE, re-ischemia events, and death, respectively, among 1,644 patients during the 5-year follow-up.

Univariate Cox regression analysis revealed that plasma exposure of statins and their metabolite have a better predictive effect on adverse cardiovascular events than dosage. However, only plasma 4-ATL exposure (HR: 1.151; 95% CI: 1.030–1.285; P = 0.0128) and plasma 4-AT exposure (HR: 1.217; 95% CI: 1.017-1.456; P = 0.0319) retained in multivariate Cox regression model, indicating that 4-ATL and 4-ATmay have more potential adverse impacts on composite clinical endpoints than AT and other metabolites. Multivariate Cox regression model also revealed that SYNTAX score (HR: 1.024; 95% CI: 1.013–1.035; P < 0.0001), heart failure (HR: 2.098; 95% CI: 1.463–3.007; P < 0.0001), CREA level (HR: 1.006; 95% CI: 1.003–1.010; P < 0.0001), TRIG level (HR: 1.114; 95% CI: 1.027–1.209; P = 0.0092), and use of proton pump inhibitors (HR: 1.446; 95% CI: 1.089–1.919; P = 0.0107) were independent risk factors for MACE (**Table 1**). A higher plasma 4-AT exposure was found to be significantly associated with increased risk of death (HR: 1.217; 95% CI: 1.079–1.495; P = 0.0319).

**Table 1:** Effects of patient characteristics on death, re-ischemia events and MACE in patients taking AT.

Characteristics	MACE				Re-ischemia events				Death			
	Univariable Analysis		Multivariable Analysis		Univariable Analysis		Multivariable Analysis		Univariable Analysis		Multivariable Analysis	
	HR (95% CI)	P Value	HR (95% CI)	P Value	HR (95% CI)	P Value	HR (95% CI)	P Value	HR (95% CI)	P Value	HR (95% CI)	P Value
**Demographic data**												
Age	1.021 (1.008–1.035)	0.0012			0.992 (0.976–1.007)	0.2980			1.084 (1.058–1.110)	<0.0001	1.054 (1.026–1.083)	0.0002
Sex (male)	1.011 (0.752–1.360)	0.9415			0.997 (0.677–1.467)	0.9859			0.988 (0.622–1.568)	0.9580		
Dosage (mg)	1.016 (1.000–1.032)	0.0567			1.011 (0.989–1.034)	0.3369			1.024 (1.000–1.048)	0.0545		
**SYNTAX score**	1.031 (1.021–1.040)	<0.0001	1.024 (1.013–1.035 )	<0.0001	1.028 (1.015–1.041)	<0.0001	1.024 (1.011–1.037)	0.0004	1.035 (1.020–1.050)	<0.0001		
**Medical history**												
Arrhythmia	1.684 (1.189–2.384)	0.0034			1.184 (0.695–2.019)	0.5343			2.421 (1.501–3.903)	0.0003		
Diabetes	1.702 (1.323–2.190)	<0.0001			1.579 (1.128–2.210)	0.0077			2.063 (1.402–3.036)	0.0002		
Heart failure	2.720 (1.983–3.731)	<0.0001	2.098 (1.463–3.007)	<0.0001	2.109 (1.330–3.345)	0.0015	1.829 (1.143–2.926)	0.0118	3.407 (2.161–5.370)	<0.0001	2.108 (1.251–3.552)	0.0051
Hypertension	1.445 (1.117–1.869)	0.0050			1.681 (1.188–2.379)	0.0033	1.605 (1.132–2.274)	0.0078	1.258 (0.848–1.867)	0.2542		
Hyperlipidemia	1.256 (0.871–1.811)	0.2228			1.586 (1.024–2.458)	0.0388	1.616 (1.040–2.511)	0.0328	0.707 (0.344–1.455)	0.3463		
**Biochemical measurements**												
ALT, U/L	1.006 (1.000–1.013)	0.0646			1.007 (0.998–1.016)	0.1336			1.006 (0.995–1.018)	0.2517		
AST, U/L	1.002 (0.999–1.005)	0.1820			1.002 (0.997–1.006)	0.4653			1.003 (0.999–1.007)	0.1566		
CREA, umol/L	1.009 (1.007–1.012)	<0.0001	1.006 (1.003–1.010)	<0.0001	1.000 (0.994–1.006)	0.9702			1.016 (1.013–1.018)	<0.0001	1.012 (1.008–1.016)	<0.0001
eGFR, ml/min/1.73 m^2^	0.991 (0.986–0.996)	0.0003			0.998 (0.995–1.002)	0.3855			0.974 (0.966–0.981)	<0.0001		
CK, U/L	1.000 (1.000–1.000)	0.0675			1.000 (1.000–1.001)	0.3281			1.000 (1.000–1.001)	0.0436	1 (1.000–1.001)	0.0015
CKMB, U/L	1.003 (0.997–1.009)	0.3043			1.001 (0.991–1.011)	0.8421			1.004 (0.996–1.013)	0.3317		
CHOL, mmol/L	1.021 (0.916–1.137)	0.7110			1.129 (0.985–1.293)	0.0806			0.838 (0.698–1.007)	0.0595		
LDLC, mmol/L	1.000 (0.876–1.142)	1.0000			1.139 (0.965–1.345)	0.1243			0.752 (0.598–0.946)	0.0147		
HDLC, mmol/L	0.615 (0.376–1.005)	0.0526			0.815 (0.435–1.529)	0.5239			0.371 (0.166–0.828)	0.0155		
TRIG, mmol/L	1.087 (1.001–1.180)	0.0473	1.114 (1.027–1.209)	0.0092	1.101 (0.994–1.220)	0.0655			1.062 (0.924–1.222)	0.3963		
GLUC, mmol/L	1.055 (1.014–1.099)	0.0084			1.044 (0.988–1.102)	0.1233			1.090 (1.029–1.155)	0.0034		
Lpa, mg/L	1.000 (1.000–1.001)	0.0916			1.000 (1.000–1.001)	0.5121			1.001 (1.000–1.001)	0.0601		
APOA, g/L	0.491 (0.288–0.836)	0.0088			0.736 (0.382–1.418)	0.3588			0.187 (0.072–0.481)	0.0005	0.258 (0.095–0.703)	0.0080
**Medication**												
*β*-blockers	1.704 (1.056–2.752)	0.0291			3.085 (1.363–6.981)	0.0069	2.608 (1.148–5.924)	0.0221	0.973 (0.534–1.776)	0.9299		
ACEIs	1.235 (0.952–1.602)	0.1118			1.564 (1.094–2.235)	0.0141			0.953 (0.642–1.414)	0.8107		
CCBs	1.341 (1.037–1.735)	0.0252			1.359 (0.970–1.906)	0.0749			1.446 (0.972–2.152)	0.0686		
PPIs	1.607 (1.250–2.065)	0.0002	1.446 (1.089–1.919)	0.0107	1.953 (1.394–2.736)	<0.0001	1.757 (1.251–2.466)	0.0011	1.221 (0.830–1.794)	0.3103		
**Plasma concentration**												
AT, ng/ml	1.113 (1.003–1.234)	0.0428			1.008 (0.883–1.151)	0.9013			1.356 (1.146–1.605)	0.0004		
2-AT, ng/ml	1.089 (0.967–1.225)	0.1598			0.948 (0.818–1.099)	0.4782			1.388 (1.141–1.688)	0.0010		
4-AT, ng/ml	1.211 (1.092–1.344)	0.0003			1.052 (0.920–1.204)	0.4584			1.548 (1.319–1.816)	<0.0001	1.217 (1.017–1.456)	0.0319
ATL, ng/ml	1.166 (1.058–1.285)	0.0019			1.069 (0.944–1.211)	0.2957			1.417 (1.211–1.659)	<0.0001		
2-ATL, ng/ml	1.200 (1.072–1.343)	0.0016			1.076 (0.933–1.241)	0.3147			1.508 (1.251–1.819)	<0.0001		
4-ATL, ng/ml	1.287 (1.157–1.431)	<0.0001	1.151 (1.030–1.285)	0.0128	1.161 (1.015–1.328)	0.0290			1.619 (1.361–1.926)	<0.0001		

Variables with P < 0.05 were entered into the multivariable model, and only variables with P < 0.05 were retained in the model.

2-AT, 2-hydroxy atorvastatin; 2-ATL, 2-hydroxy atorvastatin lactone; 4-AT, 4-hydroxy atorvastatin; 4-ATL, 4-hydroxy atorvastatin lactone; ACEIs, angiotensin converting enzyme inhibitors; ALT, alanine aminotransferase; APOA, apolipoprotein a; AST, aspartate aminotransferase; AT, atorvastatin; ATL, atorvastatin lactone; CCBs, calcium channel blockers; CHOL, cholesterol; CK, creatine kinase; CKMB, creatine kinase MB; CREA, creatinine; eGFR, estimated glomerular filtration rate; GLUC, glucose; HDLC, high-density lipoprotein cholesterol; HR, hazard ratio; LDLC, low-density lipoprotein cholesterol; Lpa, lipoprotein (a); MACEs, major adverse cardiovascular events; PPIs, proton pump inhibitors; SD, standard deviation; TRIG, triglyceride.

**Figure 2:**
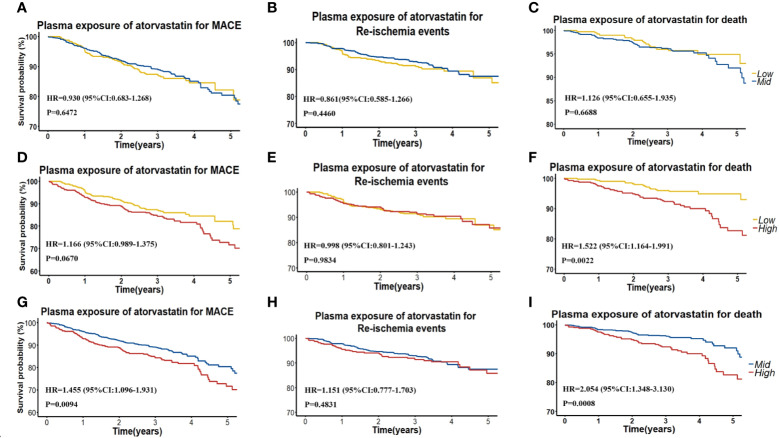
**(A–C)** Kaplan–Meier curves for cumulative incidence of MACE, re-ischemia events, and death in patients with low (<Q1) and intermediate (≥Ql and ≤Q3) plasma AT concentration. **(D–F)** Kaplan–Meier curves for cumulative incidence of MACE, re-ischemia events and death in patients with low (<Q1) and high (≥Q3) plasma AT concentration. **(G–I)** Kaplan–Meier curves for cumulative incidence of MACE, re-ischemia events, and death in patients with intermediate (≥Ql and ≤Q3) and high (≥Q3) plasma AT concentration.

To further assess the efficacy of high AT exposure versus low or moderate AT exposure on the cumulative event-free survival rate for MACE, re-ischemia events, and death, we categorized patients into groups with low (< 1.20 ng/ml), moderate (1.20–5.32 ng/ml), and high (> 5.32 ng/ml) AT concentrations (**Table 3**, **Figure 2**). No difference was found in the cumulative event-free survival rate for re-ischemia events among patients with low, moderate, and high exposures of AT or its metabolites. No significant difference was also found in the cumulative event-free survival rate for MACE and death between patients with low and moderate AT exposures. High-plasma AT exposure was associated with the increased risk of death compared with low (HR: 1.522; 95% CI: 1.035–1.061; P = 0.0022) or moderate AT exposure (HR: 2.054; 95% CI: 1.348–3.130; P = 0.0008). When we used 4-ATL concentration as a biomarker, a significantly higher risk was observed in the occurrence of MACE and death for high 4-ATL exposure versus low and moderate 4-ATL exposures with HR (95% CI) range 1.556 (1.294–1.871) to 2.710 (1.827–4.019), all P < 0.0001 (**Table 3**, **Figure 2**). Similar trends were also found in other metabolites.

### Impact of RST and Its Metabolite Exposure on MACE, Re-Ischemia Events, and Death

We further investigated whether similar adverse impacts were present on MACE and death of high exposure of another frequently used statin, that is, RST. In RST cohort, among 804 patients, we observed 113, 82, and 38 patients who experienced MACE, re-ischemia events, and death, respectively.

Both univariate and multivariate Cox regression analyses showed that a higher-plasma RST exposure was associated with a lower risk of MACE (HR: 0.911; 95% CI: 0.844–0.983; P = 0.0161) and re-ischemia events (HR: 0.897; 95% CI: 0.820–0.981; P = 0.0173) but not death (HR: 1.057; 95% CI: 0.903–1.237; P = 0.4912) (**Table 2**).

**Table 2:** Effects of patient characteristics on death, re-ischemia events and MACE in patients taking RST.

Characteristics	MACE				Re-ischemia events				Death			
	Univariable Analysis		Multivariable Analysis		Univariable Analysis		Multivariable Analysis		Univariable Analysis		Multivariable Analysis	
	HR (95% CI)	P Value	HR (95% CI)	P Value	HR (95% CI)	P Value	HR (95% CI)	P Value	HR (95% CI)	P Value	HR (95% CI)	P Value
**Demographic data**												
Age	1.030 (1.009–1.052)	0.0044	1.023 (1.002–1.045)	0.0335	1.018 (0.995–1.042)	0.1278			1.065 (1.024–1.106)	0.0015	1.048 (1.005–1.094)	0.0298
Sex (male)	1.422 (0.893–2.266)	0.1385			1.431 (0.829–2.472)	0.1985			1.286 (0.590–2.806)	0.5272		
Dosage (mg)	1.032 (0.995–1.071)	0.0877			1.045 (1.006–1.086)	0.0236	1.046 (1.004–1.090)	0.0312	0.987 (0.903–1.079)	0.7720		
**SYNTAX score**	1.027 (1.013–1.040)	<0.0001	1.019 (1.005–1.033)	0.0086	1.031 (1.015–1.046)	<0.0001	1.025 (1.009–1.042)	0.0022	1.026 (1.002–1.050)	0.0312		
**Medical history**												
Arrhythmia	1.808 (1.013–3.225)	0.0450			1.571 (0.756–3.262)	0.2260			2.526 (1.056–6.043)	0.0373		
Diabetes	1.507 (1.014–2.239)	0.0426			1.212 (0.743–1.976)	0.4414			2.066 (1.078–3.961)	0.0289		
Heart failure	2.705 (1.592–4.594)	0.0002	1.861 (1.067–3.243)	0.0285	1.669 (0.768–3.626)	0.1955			5.783 (2.808–11.911)	<0.0001	3.843 (1.755–8.415)	0.0008
Hypertension	0.890 (0.615–1.288)	0.5371			0.927 (0.601–1.432)	0.7341			0.754 (0.399–1.426)	0.3849		
Hyperlipidemia	1.198 (0.695–2.065)	0.5152			1.610 (0.904–2.867)	0.1056			0.652 (0.200–2.119)	0.4768		
**Biochemical measurements**												
ALT, U/L	1.003 (0.993–1.012)	0.5664			1.007 (0.997–1.017)	0.1560			0.992 (0.973–1.012)	0.4458		
AST, U/L	1.005 (1.001–1.009)	0.0187			1.006 (1.002–1.010)	0.0037			0.999 (0.986–1.012)	0.8644		
CREA, umol/L	1.012 (1.008–1.016)	<0.0001	1.011 (1.006–1.015)	<0.0001	1.009 (1.003–1.014)	0.0023	1.007 (1.001–1.014)	0.0245	1.018 (1.013–1.022)	<0.0001	1.019 (1.013–1.025)	<0.0001
eGFR, ml/min/1.73 m^2^	0.998 (0.994–1.002)	0.2336			1.000 (0.997–1.002)	0.8440			0.964 (0.951–0.977)	<0.0001		
CK, U/L	1.000 (1.000–1.001)	0.2183			1.000 (1.000–1.001)	0.0598			0.999 (0.996–1.002)	0.4933		
CKMB, U/L	1.011 (1.000–1.023)	0.0556			1.014 (1.002–1.026)	0.0181			0.994 (0.950–1.040)	0.7946		
CHOL, mmol/L	1.068 (0.939–1.214)	0.3192			1.165 (1.032–1.316)	0.0139			0.682 (0.490–0.948)	0.0229	0.656 (0.464–0.928)	0.0173
LDLC, mmol/L	1.149 (0.982–1.344)	0.0823			1.303 (1.116–1.521)	0.0008	1.262 (1.066–1.494)	0.0069	0.687 (0.47–1.006)	0.0534		
HDLC, mmol/L	0.608 (0.274–1.348)	0.2204			0.723 (0.288–1.812)	0.4886			0.151 (0.032–0.713)	0.0170		
TRIG, mmol/L	0.950 (0.786–1.148)	0.5967			1.031 (0.854–1.246)	0.7484			0.682 (0.419–1.109)	0.1229		
GLUC, mmol/L	1.044 (0.992–1.099)	0.0991			1.001 (0.929–1.078)	0.9888			1.107 (1.036–1.182)	0.0025		
Lpa, mg/L	1.000 (1.000–1.001)	0.1440			1.001 (1.000–1.001)	0.0349			1.000 (0.998–1.001)	0.8332		
APOA, g/L	0.635 (0.285–1.414)	0.2664			0.579 (0.228–1.468)	0.2493			0.341 (0.072–1.608)	0.1737		
**Medication**												
*β*-blockers	1.541 (0.805–2.951)	0.1917			2.872 (1.051–7.844)	0.0396			0.808 (0.338–1.933)	0.6325		
ACEIs	1.595 (1.076–2.364)	0.0202			1.670 (1.047–2.662)	0.0313			1.372 (0.709–2.652)	0.3477		
CCBs	1.576 (1.080–2.301)	0.0184			1.585 (1.017–2.471)	0.0420			1.136 (0.573–2.252)	0.7150		
PPIs	1.513 (1.034–2.213)	0.0328			1.611 (1.027–2.527)	0.0378	1.72 (1.074–2.754)	0.0240	1.166 (0.615–2.211)	0.6385		
**Plasma concentration**												
RST, ng/ml	0.912 (0.843–0.986)	0.0204	0.911 (0.844–0.983)	0.0161	0.876 (0.801–0.957)	0.0035	0.897 (0.820–0.981)	0.0173	1.057 (0.903–1.237)	0.4912		
RSTL, ng/ml	0.964 (0.857–1.083)	0.5343			0.936 (0.817–1.072)	0.3363			1.170 (0.932–1.468)	0.1761		
DM-RST, ng/ml	1.035 (0.871–1.231)	0.6936			0.930 (0.760–1.137)	0.4770			1.590 (1.177–2.149)	0.0025		

*Variables with P < 0.05 were entered into the multivariable model, and only variables with P < 0.05 were retained in the model.

DM-RST, N-desmethyl rosuvastatin; RST, rosuvastatin; RSTL, rosuvastatin lactone; other abbreviations as in **Table 1**.

**Figure 3:**
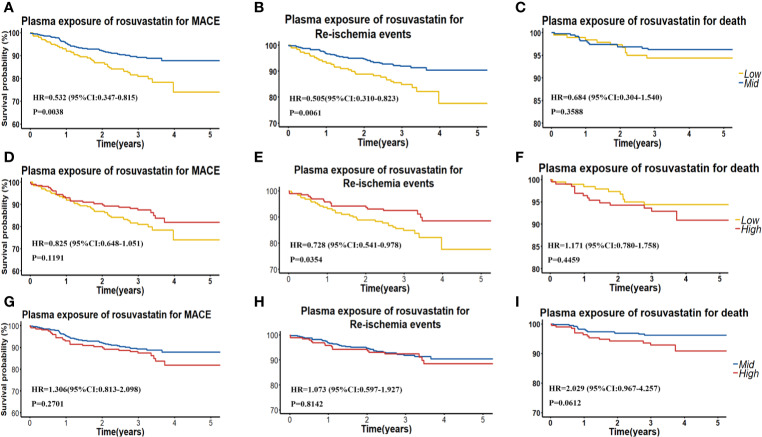
**(A–C)** Kaplan**–**Meier curves for cumulative incidence of MACE, re-ischemia events, and death in patients with low (<Q1) and intermediate (≥Ql and ≤Q3) plasma RST concentration. **(D–F)** Kaplan**–**Meier curves for cumulative incidence of MACE, re-ischemia events, and death in patients with low (< Q1) and high (≥Q3) plasma RST concentration. **(G–I)** Kaplan**–**Meier curves for cumulative incidence of MACE, re-ischemia events, and death in patients with intermediate (≥Ql and ≤Q3) and high (≥Q3) plasma RST concentration.

We also grouped patients into patients with low (< 0.53 ng/ml), moderate (0.53–4.29 ng/ml) and high (> 4.29 ng/ml) RST concentration, and we found that patients with moderate RST concentration versus low concentration had a significantly lower risk of MACE, with HR (95% CI) = 0.532 (0.347–0.815), P = 0.0038. Similarly, patients with moderate RST concentration also had a significantly lower risk of re-ischemia events, with HR (95% CI) = 0.505 (0.310–0.823), P = 0.0061. However, patients with high RSTL or DM-RST concentration *versus* moderate concentration had a significantly higher risk of death, with HR (95% CI) = 2.587 (1.244–5.378), P = 0.0109, and HR (95% CI) = 3.765 (1.738–8.158), P = 0.0008, respectively (**Table 3**, **Figure 3**).

**Table 3:** Effects of plasma exposure of statins and theirs metabolites on death, re-ischemia events and MACE in patients.

Characteristics		MACE		Re-ischemia events		Death	
		HR (95% CI)	P Value	HR (95% CI)	P Value	HR (95% CI)	P Value
**Plasma concentration in AT cohort**							
AT, ng/ml	Q1–Q3 *vs* Q1	0.930 (0.683–1.268)	0.6472	0.861 (0.585–1.266)	0.4460	1.126 (0.655–1.935)	0.6688
	Q3 *vs* Q1	1.166 (0.989–1.375)	0.0670	0.998 (0.801–1.243)	0.9834	1.522 (1.164–1.991)	0.0022
	Q3 *vs* Q1–Q3	1.455 (1.096–1.931)	0.0094	1.151 (0.777–1.703)	0.4831	2.054 (1.348–3.130)	0.0008
2-AT, ng/ml	Q1–Q3 *vs* Q1	0.995 (0.729–1.360)	0.9771	0.739 (0.505–1.083)	0.1207	1.815 (1.002–3.288)	0.0492
	Q3 *vs* Q1	1.207 (1.022–1.425)	0.0268	1.000 (0.809–1.236)	1.0000	1.765 (1.305–2.386)	0.0002
	Q3 *vs* Q1–Q3	1.455 (1.097–1.929)	0.0092	1.352 (0.913–2.001)	0.1323	1.705 (1.131–2.570)	0.0108
4-AT, ng/ml	Q1–Q3 *vs* Q1	1.061 (0.763–1.477)	0.7232	0.810 (0.545–1.205)	0.2984	2.498 (1.225–5.094)	0.0118
	Q3 *vs* Q1	1.332 (1.122–1.581)	0.0011	1.094 (0.882–1.357)	0.4133	2.227 (1.559–3.181)	<0.0001
	Q3 *vs* Q1–Q3	1.692 (1.287–2.224)	0.0002	1.477 (1.011–2.158)	0.0436	2.064 (1.386–3.074)	0.0004
ATL, ng/ml	Q1–Q3 *vs* Q1	1.161 (0.836–1.612)	0.3730	1.116 (0.744–1.672)	0.5958	1.349 (0.747–2.438)	0.3209
	Q3 *vs* Q1	1.330 (1.119–1.580)	0.0012	1.111 (0.883–1.396)	0.3695	1.806 (1.353–2.411)	<0.0001
	Q3 *vs* Q1–Q3	1.515 (1.151–1.995)	0.0030	1.107 (0.755–1.624)	0.6029	2.406 (1.591–3.636)	<0.0001
2-ATL, ng/ml	Q1–Q3 *vs* Q1	1.232 (0.886–1.712)	0.2141	1.091 (0.730–1.630)	0.6700	1.743 (0.940–3.232)	0.0776
	Q3 *vs* Q1	1.338 (1.125–1.593)	0.0010	1.104 (0.879–1.385)	0.3943	1.919 (1.412–2.607)	<0.0001
	Q3 *vs* Q1–Q3	1.451 (1.102–1.910)	0.0081	1.114 (0.759–1.636)	0.5802	2.115 (1.408–3.177)	0.0003
4-ATL, ng/ml	Q1–Q3 *vs* Q1	1.451 (1.014–2.075)	0.0415	1.212 (0.794–1.852)	0.3728	3.072 (1.384–6.817)	0.0058
	Q3 *vs* Q1	1.556 (1.294–1.871)	<0.0001	1.254 (0.993–1.582)	0.0568	2.710 (1.827–4.019)	<0.0001
	Q3 *vs* Q1–Q3	1.707 (1.307–2.229)	<0.0001	1.299 (0.896–1.884)	0.1670	2.537 (1.708–3.769)	<0.0001
**Plasma concentration in RST cohort**							
RST, ng/ml	Q1–Q3 *vs* Q1	0.532 (0.347–0.815)	0.0038	0.505 (0.310-0.823)	0.0061	0.684 (0.304–1.540)	0.3588
	Q3 *vs* Q1	0.825 (0.648–1.051)	0.1191	0.728 (0.541–0.978)	0.0354	1.171 (0.780–1.758)	0.4459
	Q3 *vs* Q1–Q3	1.306 (0.813–2.098)	0.2701	1.073 (0.597–1.927)	0.8142	2.029 (0.967–4.257)	0.0612
RSTL, ng/ml	Q1–Q3 *vs* Q1	0.597 (0.381–0.935)	0.0243	0.571 (0.341–0.957)	0.0333	0.693 (0.296–1.622)	0.3979
	Q3 *vs* Q1	1.008 (0.796–1.277)	0.9448	0.934 (0.705–1.237)	0.6334	1.328 (0.882–2.000)	0.1736
	Q3 *vs* Q1–Q3	1.708 (1.095–2.666)	0.0184	1.523 (0.893–2.598)	0.1229	2.587 (1.244–5.378)	0.0109
DM-RST, ng/ml	Q1–Q3 *vs* Q1	0.727 (0.465–1.137)	0.1620	0.781 (0.470–1.299)	0.3406	0.485 (0.202–1.164)	0.1053
	Q3 *vs* Q1	1.019 (0.799–1.299)	0.8814	0.892 (0.660–1.206)	0.4573	1.360 (0.924–2.001)	0.1194
	Q3 *vs* Q1–Q3	1.474 (0.944–2.301)	0.0881	1.068 (0.615–1.853)	0.8157	3.765 (1.738–8.158)	0.0008

Variable with P < 0.0167 was considered statistically significant.

Abbreviations as in **Tables 1** and **2**.

## Discussion

Evidence to determine the effects of statin intensity on outcomes were sparse, and recommendations were varied among guidelines (Group et al., 2008; Ridker et al., 2008; Dormuth et al., 2013). Using stratified plasma exposure to reflect the efficacy of statins might fill up the evidence gaps. Different levels of plasma statin or metabolite exposure had different impacts on clinical outcomes. To our knowledge, our study is the first to evaluate the effect of statin plasma exposure on cardiovascular event in Chinese patients with CAD. The principal findings are as follows: First, plasma exposure of statins and their metabolite have a better predictive effect on adverse cardiovascular events than dosage, thereby identifying patients with higher risks of MACE and death. Second, compared with low-plasma statin exposure, moderate exposure could achieve equal or better efficacy in terms of composite cardiovascular events, whereas high metabolite exposure of statins might increase the risk of death. Finally, 4-ATL are independently associated with increased MACE risk, and 4-AT is independently associated with death, indicating their usefulness as potential risk markers.

Our study used quantified plasma concentration, not dosage, as the measurable indicator to reflect the efficacy of statins, given that statins are subject to extensive metabolism to produce large amounts of active and toxic metabolites, whose plasma exposure linked significantly to the safety and efficacy. In addition, statin dosage did not completely represent the plasma exposure level. The same statin treatment in different individuals performed different plasma concentration, even achieved different LDL-C decrements (Reiner et al., 2013; Reiner, 2014; Kong et al., 2017). In our study, nearly 87% of patients received moderate-intensity statin treatment (20 mg of AT and 10 mg of RST), among them, 22.66–23.93% have high statin plasma concentration yet. This discrepancy might be associated with drug–drug interactions and polymorphisms in the cytochrome P450 enzymes, drug target receptors, or uptake and efflux transporter (Kim et al., 2004; Backman et al., 2005; DeGorter et al., 2013). Furthermore, statin efficacy in Asians significantly differs from that in Caucasians because of the ethnic differences and heterogeneity in response to statin (Matsuzawa et al., 2003; Birmingham et al., 2015). Therefore, using plasma concentration should be more accurate than dosage. Furthermore, given that statins present high inter-patient variability, concentration levels for statins and their metabolite should be closely monitored in patients over the course of treatment.

Excessively high plasma concentrations of highly potent statins and metabolites may not be beneficial to patients. We found that high concentrations of 4-ATL are independently associated with increased MACE risk, and 4-AT is independently associated with death, indicating that 4-ATL and 4-AT may have more potential adverse impact on composite clinical endpoints than AT and other metabolites. To address the potential confounding effect that patients who took higher statins, may have a poorer CVD risk profile and hence a higher risk of MACE, we 1) explore the effects of plasma statin exposure rather than dose on CVD, 2) in this study, 87% of patients received moderate doses of statins, however, some patients had high plasma statin exposure, which suggested that high plasma statin exposure may not entirely caused by taking high doses, possibly by inter-individual variation, 3)and we use the SYNTAX score presented the severity of CVD as a correction factor.

Further studies are necessary to determine the biological mechanism linking high plasma concentration of statins and metabolites to MACE risk. Possibly, statins impair mitochondrial function by inhibiting the synthesis of coenzyme Q10, whereas the heart depends heavily on mitochondrial ATP, which will inevitably be impaired by statin use (Nakazato et al., 2012). Among AT’s five metabolites, three lactone forms, namely ATL, 2-ATL, and 4-ATL, might be involved in toxicity (Hermann et al., 2006; Skottheim et al., 2008; Schirris et al., 2015). The pharmacologically inactive lactone forms of statins cause statin-induced myopathy and rhabdomyolysis (Hermann et al., 2006). Moreover, the lactone forms are 1,000 times more lipophilic than its acid forms and may thus contribute to an increased tissue exposure. For instance, a study of human skeletal muscle cells *in vitro* reported that statin lactone forms showed a higher potency to induce myotoxicity than the respective acid forms (Skottheim et al., 2008). Furthermore, the lactones are generally thrice more potent inducers of cytotoxicity than their corresponding acid forms (Schirris et al., 2015). Therefore, implementation of testing statin metabolite plasma concentration would allow for decreasing the risk of adverse cardiovascular events and produce the desired therapeutic effect. Overall, high exposure of statins and metabolites might be more hazardous than previously recognized, and patients should be monitored closely regarding potential cardiovascular risk. Additional research is needed to directly compare the effects of higher versus lower plasma statin concentration on clinical outcomes.

In the present study, moderate RST concentration was associated with decreased risks of MACE and re-ischemia events during the 5-year follow-up. Regarding RST, the association could be explained by a better CHOL-lowering efficacy achieved by moderate RST concentration, given the association between the degree of LDL-C lowering and decreased risk of clinical outcomes (Cholesterol Treatment Trialists C et al., 2012). Furthermore, the major metabolite of RST and DM-RST has only one-sixth to one-half the HMG-CoA reductase inhibitory activity of RST *in vitro* studies (White, 2002). Overall, approximately 90% of pharmacological activity is accounted for RST. However, in patients with AT treatment, moderate AT concentration was not statistically associated with decreased risks of MACE and re-ischemia events. Its two active metabolites, namely, 2-AT and 4-AT, are also involved in CHOL reduction (Poli, 2007; Hoffart et al., 2012), and they might, therefore, involve in cardiovascular events. Approximately 70% of the circulating inhibitory activity for HMG-CoA reductase is attributed to two active metabolites of AT, and the intrinsic efficacy of 2-AT and 4-AT is equivalent to that of AT. Thus, the cardiovascular events during AT treatment may be not influenced by the systemic AT exposure directly. As previously described, that viewpoint might be why RST exposure is more directly associated with MACE and re-ischemia events than AT exposure.

Our previous study suggests that high plasma exposure of statins may signiﬁcantly increase the risk of contrast-induced acute kidney injury (Cai et al., 2018). The risks of statin-associated muscle symptoms, acute kidney injury, and moderate or serious liver dysfunction are all heightened by increased statin doses (Hippisley-Cox and Coupland, 2010; Dormuth et al., 2013; Stroes et al., 2015). For patients who have experienced these adverse reactions, not only should the dosage be adjusted appropriately according to the patient’s conditions, but also the blood drug concentration should be monitored in real time to prevent high statin exposure. Also, clinically significant drug–drug interactions with statins are more common when an intensive statin is used (Wiggins et al., 2016). In our study, patients with high plasma concentration of statin taking PPI at the same time may have higher risks of MACE and re-ischemia. Thus, given the lack of benefit we observed for high exposure compared with moderate, low statin exposure, a reduction in dose may be warranted among patients with high plasma exposure. In addition, for patients with HF, old age and relatively high levels of CREA, special considerations are required to ensure that decisions regarding avoiding high intensive statin pharmacotherapy, which aim to maximize quality of life and reduce nonbeneficial treatments. Future research and clinical trials should aim to evaluate the efficacy and safety of statin use and plasma exposure, as well as uncover the mechanistic differences of low, intermediate and high statin plasma concentrations on affecting the risk of cardiovascular events to help inform clinical practice.

However, our study has two limitations. First, we used concentrations of statins and their metabolites at 10–12 h postdose, instead of pharmacokinetics parameter, as the representative of systemic exposure of statins. However, pharmacokinetics steady state was achieved after statin was administered at least 7 days prior to blood sampling, and this study design was in line with clinical application. Second, it was a single-center study. To address this defect, we used other cohorts that received other statin treatments to validate and confirm the impact of plasma statin and metabolite concentrations on MACE, re-ischemia events, and death. Finally, our study only provides evidence for comparisons of different plasma exposure of statin in patients, and we made no comparisons to a group of patients who were not taking statins. This is an area in need of further research. Therefore, in this setting, it is significant to examine the benefits and risks of ongoing statin use.

## Conclusions

A higher plasma exposure of AT and metabolites has a significantly higher risk of death, and moderate RST exposure has a significantly lower risk of MACE and re-ischemia events in Chinese patients with CAD. Therefore, measurement of plasma exposure of statins and metabolites might provide certain reference significance for clinically personalized medicine to optimize therapy.

## Data Availability Statement

The datasets generated for this study are available on request to the corresponding authors.

## Ethics Statement

The present study was approved by the Medical Ethical Review Committee of Guangdong General Hospital and conducted according to the Declaration of Helsinki. Written Informed consent was obtained from all individual participants included in the study.

## Author Contributions

In this project, X-HZ, L-YC, and W-HL contributed to the study design, experiment performance, follow up, and manuscript writing. S-LZ and Z-LZ contributed to study design and management and manuscript revision. XB and J-YC were involved in patient recruitment and draft revision. Y-BL, QZ, and G-DH performed the sample collection, follow-up, and data analysis. MH contributed to study design.

## Funding

This study was funded by the National Nature Science Foundation of China (No. 81872934, 81673514), National key research and development program (No. 2017YFC0909301), Guangdong key areas R & D projects, China (No. 2019B020229003).

## Conflict of Interest

The authors declare that the research was conducted in the absence of any commercial or financial relationships that could be construed as a potential conflict of interest.
