# Supplementary material for: Impact of Plasma Exposure of Statins and Their Metabolites With Major Adverse Cardiovascular Events in Chinese Patients With Coronary Artery Disease
Source: Front Pharmacol. 2020 May 27;11:675. doi: 10.3389/fphar.2020.00675 (PMC7267016; doi:10.3389/fphar.2020.00675)

Supplementary materials

Table S1 Plasma concentration distribution of atorvastatin and metabolites for the certain dose

Table S2 Plasma concentration distribution of rosuvastatin and metabolites for the certain dose

Table S3~S4 Patient characteristics and their effects on plasma concentration of AT and metabolites. Estimates were calculated by applying a linear regression model

Table S5 Patient characteristics and their effects on plasma concentration of RST and metabolites. Estimates were calculated by applying a linear regression

Figure S1 Histogram of Plasma concentration distribution of atorvastatin and metabolites for the certain dose. AT, atorvastatin; 2-AT, 2-hydroxy atorvastatin; 4-AT, 4-hydroxy atorvastatin; ATL, atorvastatin lactone; 2-ATL, 2-hydroxy atorvastatin lactone; 4-ATL, 4-hydroxy atorvastatin lactone.

Figure S2 Histogram of Plasma concentration distribution of rosuvastatin and metabolites for the certain dose. RST, rosuvastatin; RSTL, rosuvastatin lactone; DM-RST, rosuvastatin lactone.

Figure S3 Correlations between plasma concentration of AT and its metabolites 2-AT (A); 4-AT (B), ATL (C), 2-ATL (D) and 4-ATL (E). AT, atorvastatin; 2-AT, 2-hydroxy atorvastatin; 4-AT, 4-hydroxy atorvastatin; ATL, atorvastatin lactone; 2-ATL, 2-hydroxy atorvastatin lactone; 4-ATL, 4-hydroxy atorvastatin lactone.

Figure S4 Correlations between plasma concentration of RST and its metabolites DM-RST (A) and RSTL (B). RST, rosuvastatin; RSTL, rosuvastatin lactone; DM-RST, rosuvastatin lactone.

Table S1. Plasma concentration distribution of atorvastatin and metabolites for the certain dose

	**Total n=1644**					
AT and metabolites	Plasma concentration for 10mg ,ng/ml		Plasma concentration for 20mg, ng/ml		Plasma concentration for 40mg,ng/ml	
	Value n (%) 26(1.58%)	mean+SD	Value n (%) 1434(87.23%)	mean+SD	Value n (%) 184(11.19%)	mean+SD
AT						
low	15（57.69%）	0.58 ± 0.34	360（25.10%）	0.62 ± 0.35	36（19.57%）	0.51 ± 0.33
Mid	9（34.62%）	2.85 ± 0.92	743（51.81%）	2.81 ± 1.12	70（38.04%）	2.90 ± 1.11
High	2（7.69%）	5.71 ± 0.44	331（23.08%）	11.24 ± 8.91	78（42.39%）	13.24 ± 8.56
2-AT						
low	14 (53.85%)	0.66 ± 0.41	356（24.83%）	0.72 ± 0.36	41（22.28%）	0.64 ± 0.394
Mid	12（46.15%）	2.77 ± 1.09	747（52.09%）	2.80 ± 1.00	63（34.24%）	2.98 ± 1.09
High	/	/	331（23.08%）	8.63 ± 4.83	80（43.48%）	10.56 ± 7.24
4-AT						
low	9（34.62%）	0.21 ± 0.07	373（26.01%）	0.18 ± 0.10	29（15.76%）	0.16 ± 0.10

Mid	13（50.00%）	0.65 ± 0.31	736（51.32%）	0.79 ± 0.32	73（39.67%）	0.80 ± 0.30
High	4（15.38）	1.99 ± 0.36	325（22.66%）	3.99 ± 4.20	82（44.57%）	4.32 ± 3.56
ATL						
low	14（53.85%）	0.42 ± 0.254	369（25.73%）	0.47 ± 0.29	28（15.22%）	0.34 ± 0.24
Mid	10（38.46%）	1.98 ± 0.75	737（51.39%）	2.42 ± 1.01	75（40.76%）	2.67 ± 1.06
High	2（7.69%）	5.89 ± 0.30	328（22.87%）	10.66 ± 8.34	81（44.02%）	14.13 ± 13.50
2-ATL						
low	16（61.54%）	1.46 ± 0.88	365（25.45%）	1.54 ± 0.85	30（16.30%）	1.12 ± 0.75
Mid	9（34.62%）	6.38 ± 2.66	737（51.39%）	6.55 ± 2.51	76（41.30%）	6.83 ± 2.60
High	1（3.85%）	13.07 ± 0	332（23.15%）	21.63 ± 11.63	78（42.39%）	28.20 ± 14.51
4-ATL						
low	10（38.46%）	0.28 ± 0.13	377（26.29%）	0.24 ± 0.15	24（13.04%）	0.19 ± 0.16
Mid	14（53.85%）	0.90 ± 0.38	727（50.70%）	1.07 ± 0.41	81（44.02%）	1.12 ± 0.41
High	2（7.69%）	3.58 ± 2.12	330（23.01%）	4.55 ± 4.153	79（42.93%）	5.11 ± 3.52

*AT= atorvastatin;2-AT = 2-hydroxy atorvastatin; 4-AT = 4-hydroxy atorvastatin; 2-ATL = 2-hydroxy atorvastatin lactone; 4-ATL = 4-hydroxy atorvastatin lactone; ATL = atorvastatin lactone

Table S2. **Plasma concentration distribution of rosuvastatin and metabolites for the certain dose**

RST and metabolites	Total n=804							
	Plasma concentration for 5mg ,ng/ml		Plasma concentration for 10mg, ng/ml		Plasma concentration for 20mg,ng/ml		Plasma concentration for 40mg,ng/ml	
	Value n (%) 16(1.99%)	mean+SD	Value n (%) 702(87.31%)	mean+SD	Value n (%) 80(9.95%)	mean+SD	Value n (%) 6(0.75%)	mean+SD
RST								
low	3（18.75%）	0.02 ± 0.02	176（25.07%）	0.08 ± 0.13	19（23.75%）	0.05 ± 0.08	3（50.00%）	0.03 ± 0.01
Mid	10（62.50%）	1.72 ± 0.48	362（51.57%）	2.29 ± 1.03	29（36.25%）	2.40 ± 1.01	1（16.67%）	2.74 ± 0
High	3（18.75%）	7.97 ± 1.76	164（23.36%）	7.91 ± 3.65	32（40.00%）	10.59 ± 7.50	2（33.33%）	7.81 ± 1.05
RSTL								
low	4（25.00%）	0.03 ± 0.02	174（24.79%）	0.02 ± 0.01	19（23.75%）	0.02 ± 0.01	4（66.67%）	0.02 ± 0.01
Mid	7（43.75%）	0.22 ± 0.12	364（51.85%）	0.30 ± 0.13	30（37.50%）	0.33 ± 0.14	1（16.67%）	0.49 ± 0
High	5（31.25%）	1.21 ± 0.69	164（23.36%）	1.07 ± 0.58	31（38.75%）	1.45 ± 0.79	1（16.67%）	1.81 ± 0
DMRST								

low	4（25.00%）	0.05 ± 0.03	176（25.07%）	0.07 ± 0.02	20（25.00%）	0.07 ± 0.01	1（16.67%）	0.07 ± 0
Mid	5（31.25%）	0.20 ± 0.06	358（51.00%）	0.26 ± 0.12	35（43.75%）	0.31 ± 0.14	4（66.67%）	0.20 ± 0.10
High	7（43.75%）	0.68 ± 0.17	168（23.93%）	1.09 ± 0.74	25（31.25%）	1.79 ± 3.02	1（16.67%）	0.87 ± 0

*DM-RST = N-desmethyl rosuvastatin; RST = rosuvastatin; RSTL = rosuvastatin lactone

Table S3. Patient characteristics and their effects on plasma concentrations of AT, 2-AT and 4-AT. ~~**Estimates were calculated by applying a linear regression model.**~~

Characteristics		Value N (%) or mean ± SD	Plasma AT concentration, ng/mL					Plasma 2-AT concentration, ng/mL					Plasma 4-AT concentration, ng/mL				
				Univariable Analysis		Multivariable Analysis			Univariable Analysis		Multivariable Analysis			Univariable Analysis		Multivariable Analysis	
			mean ± SD	Estimate	p-value	Estimate	p-value	mean ± SD	Estimate	p-value	Estimate	p-value	mean ± SD	Estimate	p-value	Estimate	p-value
**Demographic data**																	
Total number		1644	4.46 ± 6.16					3.84 ± 4.19					1.45 ± 2.54				
Age (years)		62.74 ± 9.98		0.0118	< 0.0001	0.0140	0.0002		0.0118	< 0.0001	0.0146	< 0.0001		0.0162	< 0.0001	0.0172	< 0.0001
Sex	Female	372 (22.63)	4.89 ± 7.06	0.0090	0.9009			4.31 ± 5.23	-0.0719	0.2565			1.62 ± 3.23	-0.0152	0.8347		
	Male	1272 (77.37)	4.33 ± 5.87					3.70 ± 3.82					1.40 ± 2.30				
Dosage (mg)	10	26 (1.58)	1.76 ± 1.69	0.0217	< 0.0001	0.0219	< 0.0001	1.63 ± 1.32	0.0178	< 0.0001	0.0193	< 0.0001	0.70 ± 0.64	0.0265	< 0.0001	0.0257	< 0.0001
	20	1434 (87.23)	4.20 ± 5.89					3.63 ± 3.76					1.35 ± 2.48				
	40	184 (11.19)	6.81 ± 7.91					5.75 ± 6.46					2.27 ± 3.02				
**SYNTAX score**		14.47±11.75		0.0082	0.0014				0.0057	0.0122				0.0109	<.0001		
**Medical history**																	
Arrhythmia	No	1485 (90.71)	4.38 ± 5.79	-0.0713	0.4921			3.78 ± 4.03	-0.0262	0.7745			1.42 ± 2.42	0.0914	0.3838		
	Yes	152 (9.29)	4.98 ± 8.40					4.18 ± 4.94					1.74 ± 3.52				
Diabetes	No	1214 (74.16)	4.37 ± 6.06	-0.0148	0.8301			3.74 ± 4.04	0.0312	0.6069			1.30 ± 2.22	0.3142	< 0.0001	0.2802	< 0.0001
	Yes	423 (25.84)	4.63 ± 6.13					4.06 ± 4.35					1.86 ± 3.27				
Heart failure	No	1500 (91.63)	4.35 ± 5.87	-0.0556	0.6092			3.78 ± 3.88	-0.1392	0.1459			1.38 ± 2.36	0.1875	0.0883		
	Yes	137 (8.37)	5.40 ± 8.00					4.31 ± 6.23					2.20 ± 3.97				
Hypertension	No	688 (41.98)	4.25 ± 5.97	0.0398	0.5147			3.71 ± 3.74	-0.0045	0.9338			1.33 ± 2.49	0.1128	0.0675		
	Yes	951 (58.02)	4.62 ± 6.31					3.93 ± 4.48					1.53 ± 2.58				
Hyperlipidemia	No	1452 (88.59)	4.52 ± 6.37	0.0397	0.6758			3.84 ± 4.06	0.0098	0.9062			1.47 ± 2.57	-0.0856	0.3719		
	Yes	187 (11.41)	4.03 ± 4.34					3.83 ± 5.08					1.25 ± 2.32				

**Biochemical measurements**																	
ALT, U/L		29.06 ± 17.28		0.0067	0.0003	0.0059	0.0041		0.0039	0.0169	0.0039	0.0358		0.0061	0.0010	0.0056	0.0062
AST, U/L		30.96 ± 29.74		0.0040	< 0.0001				0.0027	0.0025				0.0033	0.0015		
CREA, umol/L		87.85 ± 32.56		0.0043	< 0.0001	0.0026	0.0195		0.0024	0.0040				0.0063	< 0.0001	0.0046	< 0.0001
eGFR, ml/min/1.73 m^2		95.04 ± 77.73		-0.0010	0.0126				-0.0005	0.1686				-0.0008	0.0353		
CK, U/L		152.66 ± 380.24		0.0000	0.5627				0.0000	0.7453				-0.0001	0.3685		
CKMB, U/L		8.77 ± 14.29		0.0035	0.1252				0.0029	0.1391				0.0020	0.3690		
CHOL, mmol/L		4.31 ± 1.13		0.0806	0.0028				0.0495	0.0377				-0.0970	0.0004	-0.09269	0.0084
LDLC, mmol/L		2.60 ± 0.93		0.1178	0.0003	0.1199	0.0016		0.0789	0.0060				-0.0751	0.0232		
HDLC, mmol/L		0.98 ± 0.26		-0.3281	0.0057				-0.0411	0.6953				-0.4712	< 0.0001		
TRIG, mmol/L		1.61 ± 1.08		0.0427	0.1307				-0.0071	0.7760				-0.0880	0.0022	-0.07498	0.0388
GLUC, mmol/L		6.63 ± 2.60		0.0199	0.0887				0.0196	0.0566				0.0548	< 0.0001	0.05257	0.0001
Lpa, mg/L		288.79 ± 312.66		0.0002	0.0947				0.0001	0.1707				0.0000	0.9914		
APOA, g/L		1.05 ± 0.28		-0.5130	< 0.0001	-0.4192	0.0011		-0.2969	0.0061	-0.24401	0.0309		-0.5855	< 0.0001	-0.42177	0.0015
**Medication**																	
β-blockers	No	185 (11.29)	3.92 ± 4.56	0.0501	0.5986			3.43 ± 3.24	0.1075	0.2002			1.15 ± 1.56	0.1757	0.0671		
	Yes	1453 (88.71)	4.49 ± 6.13					3.88 ± 4.27					1.48 ± 2.61				
ACEIs	No	639 (39.01)	4.53 ± 6.24	-0.0140	0.8209			3.94 ± 4.20	-0.0281	0.6059			1.40 ± 2.23	0.0979	0.1161		
	Yes	999 (60.99)	4.36 ± 5.81					3.75 ± 4.15					1.46 ± 2.68				
CCBs	No	1184 (72.28)	4.25 ± 5.49	0.0347	0.6058			3.78 ± 3.75	-0.0309	0.6022			1.38 ± 2.32	0.0723	0.2870		
	Yes	454 (27.72)	4.89 ± 7.08					3.95 ± 5.09					1.60 ± 2.95				

PPIs	No	837 (51.1)	4.02 ± 5.53	0.0909	0.1314	3.62 ± 3.80	0.0382	0.4723	1.31 ± 2.36	0.1163	0.0555
	Yes	801 (48.9)	4.85 ± 6.39			4.04 ± 4.51			1.57 ± 2.66		

*Variables with P < 0.05 were entered into the multivariable model, and only variables with P < 0.05 were retained in the model.

‡2-AT = 2-hydroxy atorvastatin; 4-AT = 4-hydroxy atorvastatin; ACEIs = angiotensin converting enzyme inhibitors; ALT = alanine aminotransferase; APOA = apolipoprotein a; AST = aspartate aminotransferase; AT = atorvastatin; CCBs = calcium channel blockers; CHOL = cholesterol; CK = creatine kinase; CKMB = creatine kinase MB; eGFR = estimated glomerular filtration rate; GLUC = glucose; HDLC = high-density lipoprotein cholesterol; LDLC = low-density lipoprotein cholesterol; Lpa = lipoprotein (a); PPIs = proton pump inhibitors; Scr = serum creatinine; SD = standard deviation; TRIG = triglyceride.

Table S4. Patient characteristics and their effects on plasma concentrations of ATL, 2-ATL and 4-ATL. ~~Estimates were calculated by applying a linear regression model.~~

Characteristics		Value N (%) or mean ± SD	Plasma ATL concentration, ng/mL					Plasma 2-ATL concentration, ng/mL					Plasma 4-ATL concentration, ng/mL				
				Univariable Analysis		Multivariable Analysis			Univariable Analysis		Multivariable Analysis			Univariable Analysis		Multivariable Analysis	
			mean ± SD	Estimate	p-value	Estimate	p-value	mean ± SD	Estimate	p-value	Estimate	p-value	mean ± SD	Estimate	p-value	Estimate	p-value
**Demographic data**																	
Total number		1644	4.16 ± 6.44					9.37 ± 10.35					1.76 ± 2.66				
Age (years)		62.74 ± 9.98		0.0157	< 0.0001	0.0170	< 0.0001		0.0152	< 0.0001	0.0168	< 0.0001		0.0199	< 0.0001	0.0211	< 0.0001
Sex	Female	372 (22.63)	3.85 ± 5.60	0.1345	0.0871			9.78 ± 11.15	0.0118	0.8637			1.77 ± 2.94	0.1000	0.1962		
	Male	1272 (77.37)	4.25 ± 6.67					9.26 ± 10.10					1.76 ± 2.57				
Dosage (mg)	10	26 (1.58)	1.44 ± 1.59	0.0302	< 0.0001	0.0294	< 0.0001	3.61 ± 3.47	0.0248	< 0.0001	0.0253	< 0.0001	0.87 ± 0.99	0.0275	< 0.0001	0.0266	< 0.0001
	20	1434 (87.23)	3.80 ± 5.57					8.76 ± 9.43					1.65 ± 2.58				
	40	184 (11.19)	7.36 ± 10.81					14.96 ± 15.00					2.71 ± 3.13				
**SYNTAX score**		14.47±11.75		0.0083	0.0029				0.006	0.014				0.0158	<.0001	0.00841	0.0113
**Medical history**																	
Arrhythmia	No	1485 (90.71)	4.11 ± 6.26	0.0230	0.8393			9.26 ± 10.13	0.0486	0.6230			1.73 ± 2.53	0.1878	0.0933		
	Yes	152 (9.29)	4.54 ± 7.91					10.19 ± 11.71					2.04 ± 3.67				
Diabetes	No	1214 (74.16)	4.12 ± 6.26	-0.0796	0.2899			9.18 ± 10.07	-0.0047	0.9429			1.68 ± 2.67	0.2465	0.0009	0.2153	0.0049
	Yes	423 (25.84)	4.25 ± 6.91					9.84 ± 10.85					1.98 ± 2.62				
Heart failure	No	1500 (91.63)	4.07 ± 6.41	0.0919	0.4398			9.21 ± 10.09	-0.0088	0.9325			1.67 ± 2.43	0.1914	0.1026		
	Yes	137 (8.37)	5.10 ± 6.60					10.87 ± 12.08					2.65 ± 4.34				
Hypertension	No	688 (41.98)	4.30 ± 7.03	-0.0106	0.8737			9.38 ± 10.21	-0.0147	0.8002			1.73 ± 2.62	0.1294	0.0491		
	Yes	951 (58.02)	4.07 ± 6.00					9.37 ± 10.46					1.78 ± 2.69				
Hyperlipidemia	No	1452 (88.59)	4.28 ± 6.74	-0.0364	0.7254			9.53 ± 10.65	0.0129	0.8864			1.81 ± 2.78	-0.1016	0.3197		

	Yes	187 (11.41)	3.25 ± 3.31					8.17 ± 7.53					1.34 ± 1.41				
**Biochemical measurements**																	
ALT, U/L		29.06 ± 17.28		0.0074	0.0003				0.0047	0.0078				0.0075	0.0002		
AST, U/L		30.96 ± 29.74		0.0057	< 0.0001	0.0049	0.0004		0.0042	< 0.0001	0.0035	0.0048		0.0050	< 0.0001	0.0047	0.0004
CREA, umol/L		87.85 ± 32.56		0.0073	< 0.0001	0.0045	0.0002		0.0048	< 0.0001	0.0027	0.0158		0.0070	< 0.0001	0.0046	< 0.0001
eGFR, ml/min/1.73 m2		95.04 ± 77.73		-0.0021	< 0.0001				-0.0014	0.0003				-0.0020	< 0.0001		
CK, U/L		152.66 ± 380.24		0.0002	0.0653				0.0001	0.1989				0.0001	0.1950		
CKMB, U/L		8.77 ± 14.29		0.0059	0.0160				0.0050	0.0206				0.0047	0.0570		
CHOL, mmol/L		4.31 ± 1.13		0.0943	0.0014				0.0651	0.0114				-0.0607	0.0371	-0.06913	0.0455
LDLC, mmol/L		2.60 ± 0.93		0.1390	< 0.0001	0.15057	0.0006		0.0989	0.0015	0.09377	0.0158		-0.0468	0.1833		
HDLC, mmol/L		0.98 ± 0.26		-0.3386	0.0093				-0.0272	0.8105				-0.5224	< 0.0001		
TRIG, mmol/L		1.61 ± 1.08		0.0171	0.5804				-0.0213	0.4295				-0.0555	0.0691		
GLUC, mmol/L		6.63 ± 2.60		0.0163	0.2027				0.0135	0.2260				0.0469	0.0002	0.05412	0.0002
Lpa, mg/L		288.79 ± 312.66		0.0003	0.0045				0.0003	0.0053				0.0003	0.0084		
APOA, g/L		1.05 ± 0.28		-0.6067	< 0.0001	-0.50501	0.0004		-0.3508	0.0030	-0.2865	0.0247		-0.6714	< 0.0001	-0.46428	0.001
**Medication**																	
β-blockers	No	185 (11.29)	3.64 ± 4.56	0.0451	0.6646			8.54 ± 8.22	0.0357	0.6938			1.59 ± 1.83	0.0957	0.3501		
	Yes	1453 (88.71)	4.21 ± 6.58					9.45 ± 10.51					1.77 ± 2.70				
ACEIs	No	639 (39.01)	4.01 ± 5.49	0.0646	0.3387			9.27 ± 9.89	0.0275	0.6408			1.68 ± 2.52	0.1429	0.0316		
	Yes	999 (60.99)	4.23 ± 6.90					9.39 ± 10.53					1.79 ± 2.68				
CCBs	No	1184 (72.28)	4.05 ± 6.32	0.0784	0.2863			9.22 ± 9.87	0.0293	0.6480			1.71 ± 2.55	0.1328	0.0667		

	Yes	454 (27.72)	4.38 ± 6.55			9.66 ± 11.29			1.85 ± 2.79		
PPIs	No	837 (51.1)	3.69 ± 5.21	0.1335	0.0426	8.67 ± 9.18	0.0755	0.1886	1.59 ± 2.37	0.1903	0.0033
	Yes	801 (48.9)	4.61 ± 7.39			10.05 ± 11.28			1.90 ± 2.84		

*Variables with $P < 0.05$ were entered into the multivariable model, and only variables with $P < 0.05$ were retained in the model.

†2-ATL = 2-hydroxy atorvastatin lactone; 4-ATL = 4-hydroxy atorvastatin lactone; ATL = atorvastatin lactone; other abbreviations as in Table S3.

Table S5. Patient characteristics and their effects on plasma concentration of RST, RSTL and DM-RST. ~~**Estimates were calculated by applying a linear regression model.**~~

Characteristics		Value N (%) or mean ± SD	Plasma RST concentration, ng/mL					Plasma RSTL concentration, ng/mL					Plasma DM-RST concentration, ng/mL				
				Univariable Analysis		Multivariable Analysis			Univariable Analysis		Multivariable Analysis			Univariable Analysis		Multivariable Analysis	
			mean ± SD	Estimate	p-value	Estimate	p-value	mean ± SD	Estimate	p-value	Estimate	p-value	mean ± SD	Estimate	p-value	Estimate	p-value
**Demographic data**																	
Total number		804	3.25 ± 3.89					0.44 ± 0.53					0.44 ± 0.77				
Age (years)		62.37 ± 9.70		0.0019	0.8099				0.0065	0.2567				0.0102	0.0101	0.0096	0.0151
Sex	Female	200 (24.88)	3.75 ± 4.83	-0.1100	0.5368			0.50 ± 0.57	-0.0850	0.5101			0.53 ± 1.24	-0.0859	0.3344		
	Male	604 (75.12)	3.08 ± 3.51					0.42 ± 0.51					0.41 ± 0.52				
Dosage (mg)	5	16 (1.99)	2.57 ± 2.86	0.0068	0.7249			0.48 ± 0.63	0.0060	0.6703			0.37 ± 0.31	0.0138	0.1539		
	10	702 (87.31)	3.05 ± 3.42					0.41 ± 0.48					0.41 ± 0.54				
	20	80 (9.95)	5.12 ± 6.59					0.69 ± 0.79					0.71 ± 1.82				
	40	6 (0.75)	3.07 ± 3.85					0.40 ± 0.72					0.29 ± 0.30				
**SYNTAX score**		14.16±12.41		0.001804	0.7744				0.0126	0.0056				0.0053	0.0888		
**Medical history**																	
Arrhythmia	No	745 (92.66)	3.28 ± 3.94	-0.6050	0.0403			0.44 ± 0.53	-0.3567	0.0953			0.45 ± 0.79	-0.2590	0.0791		
	Yes	59 (7.34)	2.81 ± 3.18					0.41 ± 0.52					0.36 ± 0.42				
Diabetes	No	599 (74.5)	3.05 ± 3.31	-0.0940	0.5949			0.42 ± 0.49	-0.0542	0.6722			0.39 ± 0.45	0.1376	0.1191		
	Yes	205 (25.5)	3.81 ± 5.19					0.49 ± 0.63					0.60 ± 1.30				
Heart failure	No	751 (93.41)	3.10 ± 3.44	-0.0517	0.8677			0.43 ± 0.50	0.0908	0.6865			0.41 ± 0.50	0.3445	0.0261	0.3641	0.0297
	Yes	53 (6.59)	5.33 ± 7.61					0.63 ± 0.83					0.94 ± 2.28				
Hypertension	No	365 (45.4)	3.08 ± 3.43	-0.0782	0.6132			0.43 ± 0.53	-0.0178	0.8738			0.41 ± 0.54	0.0650	0.4005		
	Yes	439 (54.6)	3.38 ± 4.23					0.44 ± 0.53					0.47 ± 0.91				
Hyperlipidemia	No	707 (87.94)	3.24 ± 3.88	-0.2954	0.2113			0.43 ± 0.50	-0.0522	0.7606			0.45 ± 0.80	-0.1690	0.1524		

	Yes	97 (12.06)	3.29 ± 3.96					0.49 ± 0.71					0.39 ± 0.53				
**Biochemical measurements**																	
ALT, U/L		30.16 ± 19.47		0.0006	0.8932				0.0047	0.1134				-0.0020	0.3423		
AST, U/L		30.94 ± 27.71		-0.0082	0.0033				-0.0009	0.6680				-0.0018	0.2013		
CREA, umol/L		86.86 ± 31.99		-0.0010	0.6935				0.0029	0.0928				0.0020	0.1011		
eGFR, ml/min/1.73 m^2		100.66 ± 95.81		0.0008	0.3377				-0.0003	0.5889				-0.0002	0.6865		
CK, U/L		133.87 ± 250.07		-0.0011	0.0004	-0.0011	0.0005		-0.0002	0.3327				-0.0002	0.2411		
CKMB, U/L		7.81 ± 9.11		-0.0239	0.0052				0.0002	0.9737				-0.0003	0.9466		
CHOL, mmol/L		4.48 ± 1.30		-0.1476	0.0144				-0.0206	0.6355				-0.0799	0.0081		
LDLC, mmol/L		2.75 ± 1.06		-0.2425	0.0011	-0.2589	0.0005		-0.0424	0.4279				-0.1030	0.0056	-0.0893	0.0313
HDLC, mmol/L		1.01 ± 0.25		-0.3201	0.3059				-0.0790	0.7250				-0.4451	0.0044		
TRIG, mmol/L		1.62 ± 1.11		-0.0131	0.8527				0.0084	0.8688				-0.0145	0.6825		
GLUC, mmol/L		6.79 ± 2.98		-0.0232	0.3692				0.0027	0.8863				0.0068	0.6003		
Lpa, mg/L		266.70 ± 290.59		0.0000	0.8960				0.0003	0.1268				0.0000	0.9135		
APOA, g/L		1.09 ± 0.28		-0.1011	0.7451				-0.4255	0.0630				-0.4536	0.0037	-0.3947	0.0117
**Medication**																	
β-blockers	No	102 (12.69)	3.12 ± 3.47	-0.0470	0.8390			0.40 ± 0.48	0.1918	0.2525			0.38 ± 0.49	0.0449	0.6976		
	Yes	702 (87.31)	3.26 ± 3.95					0.44 ± 0.54					0.45 ± 0.80				

ACEIs	No	346 (43.03)	3.23 ± 3.29	-0.2217	0.1538			0.43 ± 0.53	0.1227	0.2760			0.43 ± 0.52	-0.0919	0.2369	
	Yes	458 (56.97)	3.26 ± 4.28					0.45 ± 0.53					0.45 ± 0.91			
CCBs	No	564 (70.15)	3.31 ± 4.06	-0.4080	0.0152	-0.4789	0.0055	0.44 ± 0.54	-0.0189	0.8771			0.45 ± 0.86	-0.0395	0.6387	
	Yes	240 (29.85)	3.09 ± 3.46					0.44 ± 0.50					0.42 ± 0.48			
PPIs	No	385 (47.89)	3.06 ± 3.27	-0.0692	0.6537			0.45 ± 0.53	-0.0513	0.6461			0.40 ± 0.49	0.0490	0.5250	
	Yes	419 (52.11)	3.42 ± 4.38					0.42 ± 0.53					0.48 ± 0.95			

*Variables with P < 0.05 were entered into the multivariable model, and only variables with P < 0.05 were retained in the model.

‡DM-RST = N-desmethyl rosuvastatin; RST = rosuvastatin; RSTL = rosuvastatin lactone; other abbreviations as in **Table S3**.

Figure S1. Histogram of Plasma concentration distribution of atorvastatin and metabolites for the certain dose. AT, atorvastatin; 2-AT, 2-hydroxy atorvastatin; 4-AT, 4-hydroxy atorvastatin; ATL, atorvastatin lactone; 2-ATL, 2-hydroxy atorvastatin lactone; 4-ATL, 4-hydroxy atorvastatin lactone.

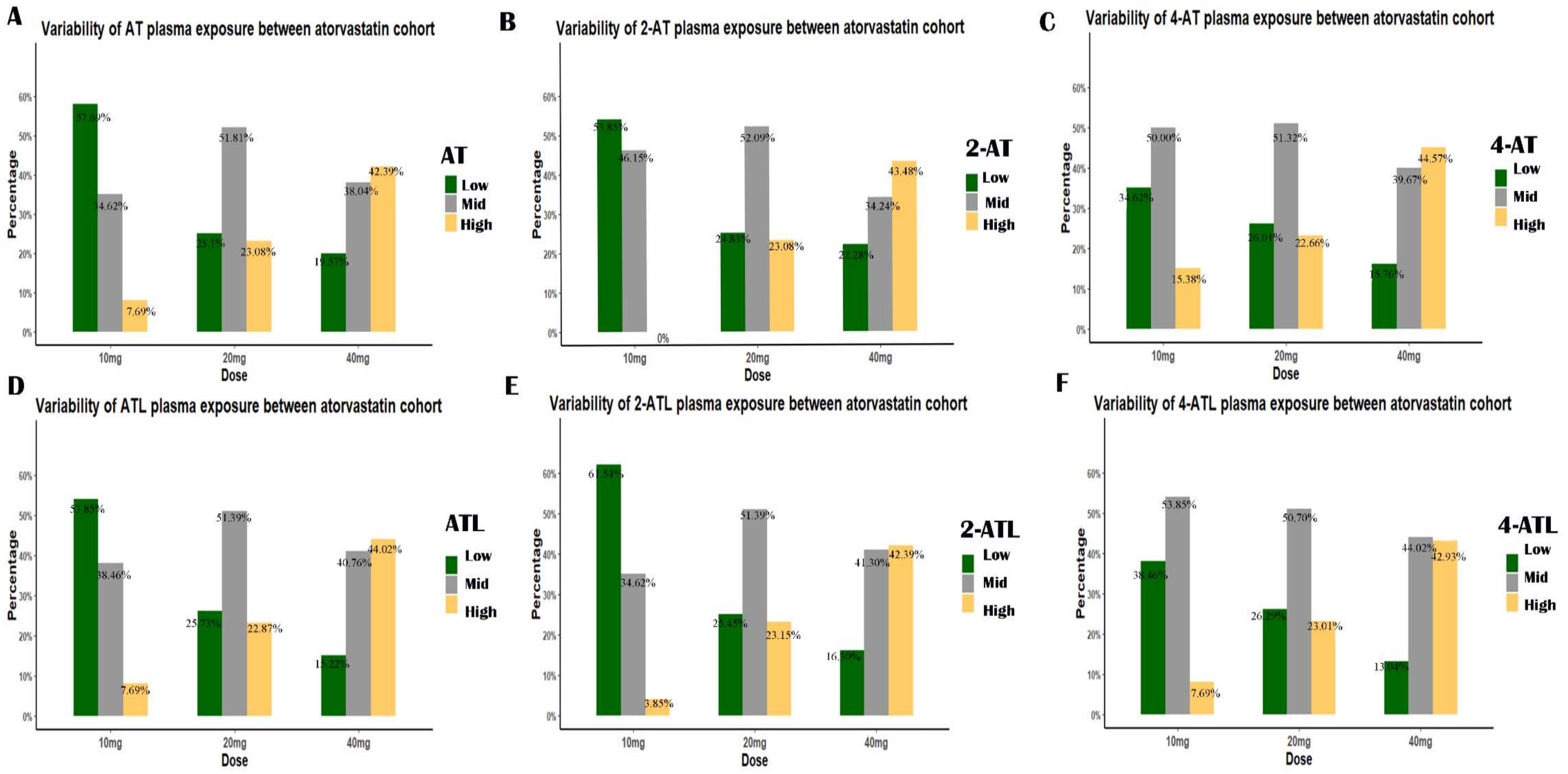

Figure S2. Histogram of Plasma concentration distribution of rosuvastatin and metabolites for the certain dose. RST, rosuvastatin; RSTL, rosuvastatin lactone; DM-RST, rosuvastatin lactone.

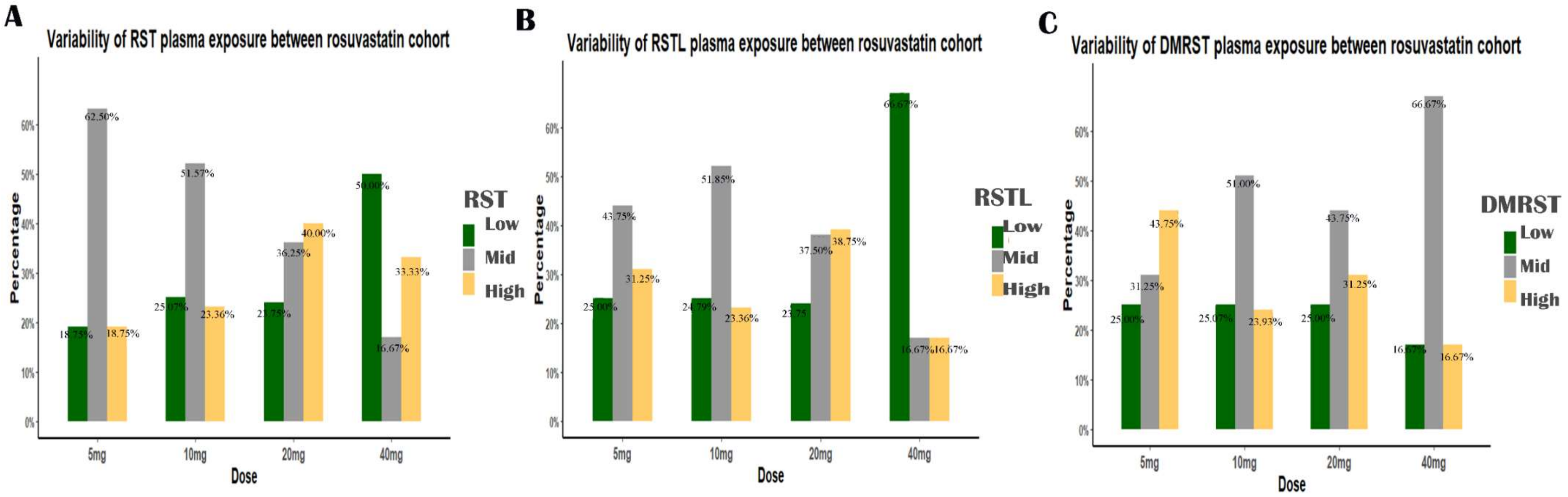

Figure S3 Correlations between plasma concentration of AT and its metabolites 2-AT (A); 4-AT (B), ATL (C), 2-ATL (D) and 4-ATL (E). AT, atorvastatin; 2-AT, 2-hydroxy atorvastatin; 4-AT, 4-hydroxy atorvastatin; ATL, atorvastatin lactone; 2-ATL, 2-hydroxy atorvastatin lactone; 4-ATL, 4-hydroxy atorvastatin lactone.

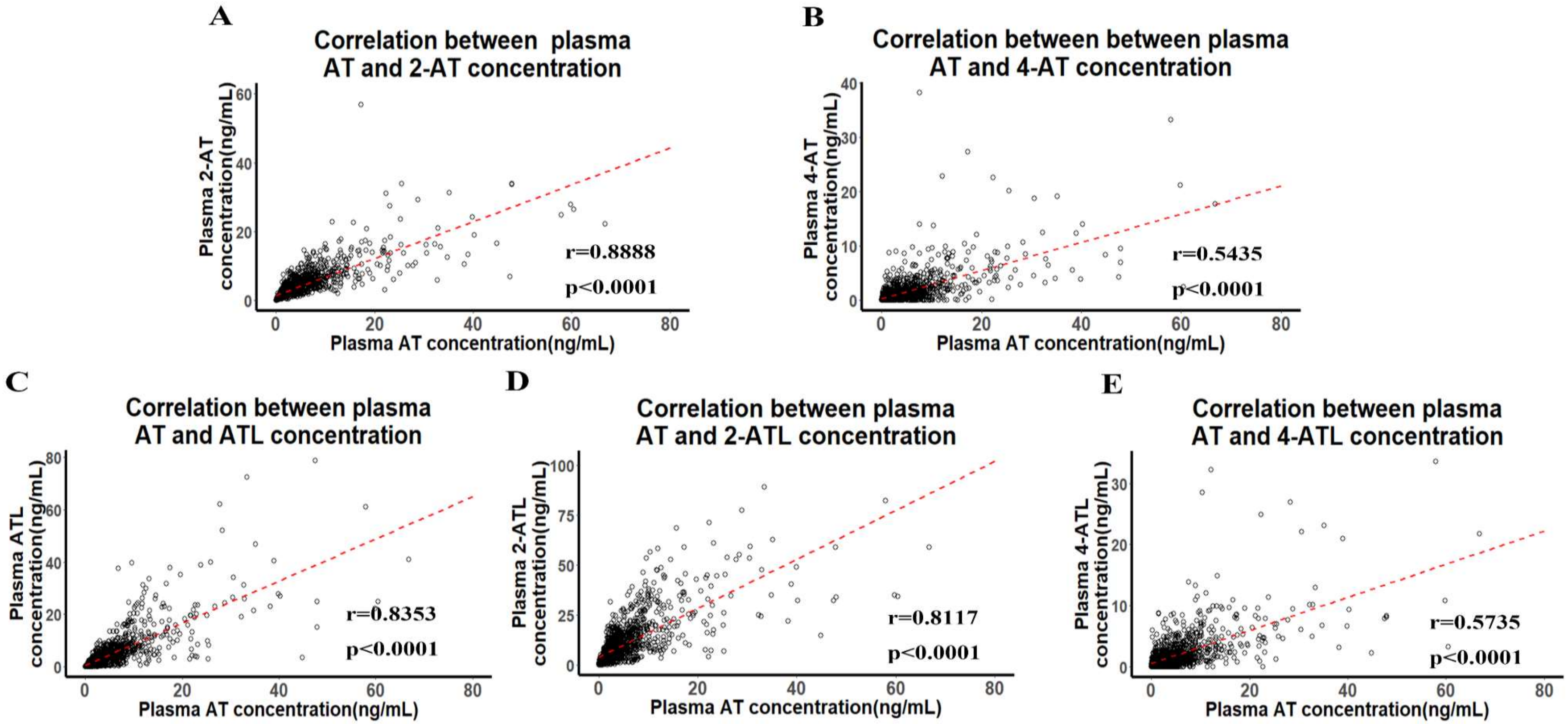

Figure S4 Correlations between plasma concentration of RST and its metabolites DM-RST (A) and RSTL (B). RST, rosuvastatin; RSTL, rosuvastatin lactone; DM-RST, rosuvastatin lactone.

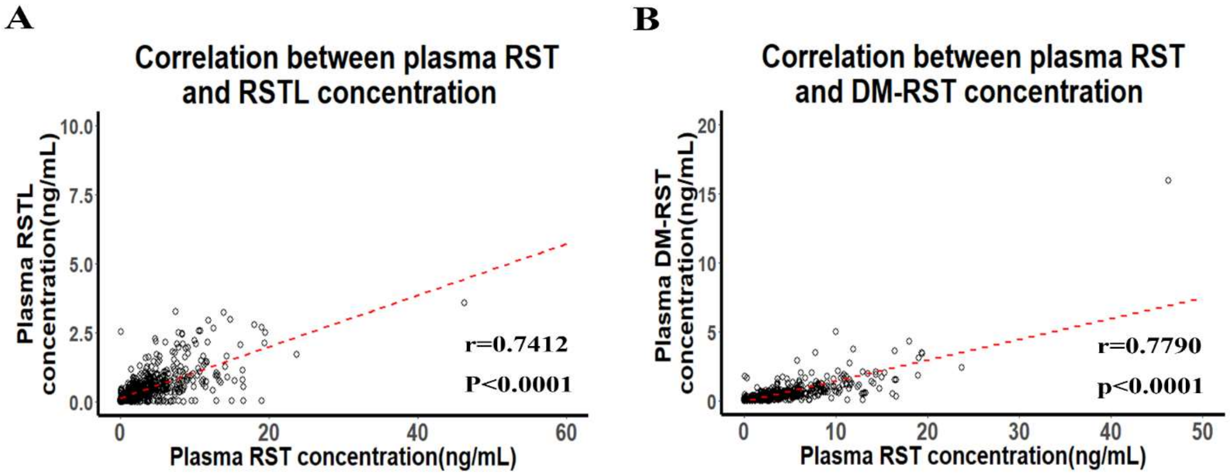

Supplement: Supplementary file 1 [file DataSheet_1.pdf]
